# Transactions, Mississippi Valley Dental Association

**Published:** 1877-05

**Authors:** 


### TRANSACTIONS OF THE MISSISSIPPI VALLEY DENTAL ASSOCIATION.

### DISCUSSION.

[Continued from page 167.]

*Thursday Morning, March, 8th.*

The subject of Mechanical Dentistry was announced for consideration, (Dr. Frank Hunter) said he would like to revive an interest in a matter almost forgotten by the profession—at least much neglected and fallen into disuse. The matter of pivoting teeth. He would protest against the wholesale extraction of the roots especially of front teeth. There are hundreds of cases where the insertion of a plate for a single tooth could be avoided, by the exercise of a little ingenuity and patience in devising means for pivoting the root, even where the indications are not such as to directly suggest such an expedient. Even the old fashioned wooden pivots have often lasted for a long time, and were worthy of being employed more generally than was practiced. By the new and improved methods, teeth may be pivoted so as to give reasonable promise of enduring for from five to twenty years.

Dr. Hunter then explained by means of models, a case of a bicuspid of which the outer cusp and nearly half the crown had been split off, upon this he had attached a porcelain crown by means of a metallic pivot inserted into the root which was first filled with amalgam and then drilled to accommodate the pivot, and a metallic band platinum which encircled the remaining portion of the crown. The operation was very neatly performed.

Dr. Keely. I second Dr. Hunter's protest against the wholesale extraction of anterior roots, especially where there are two or three roots only to be restored to a servicable condition. My attention was first directed to the importance of pivoting, thirty-four years ago in this city, at Dr. Rogers' office. He showed me two pivoted central incisors in the mouth of a lady for whom he had performed the operation eighteen years before. He was very enthusiastic over it.

In 1874, I saw a person for whom I had inserted three pivot teeth in 1842. Two of these I had re-set, but the third was there still on the original pivot.

A gentleman for whom I had inserted two superior incisors twenty-two years before, showed them to me recently just as firm and solid as when they were put in. Stockton's pivot teeth were used. They were better than those made now-a-days but not of so good color.

No one ever wore a single tooth on an atmospheric plate with any degree of comfort. The old theory was that pivot teeth last from three to five years. Even upon that reckoning I think it is our duty to insert them where we can.

Dr. Waye. Were those pivots made of the ordinary compressed hickory?

Dr. Keely. Yes sir. My plan has always been to envelope the pivot in gold foil. Sometimes I use os-artificial, and the tooth lasts four or five years.

Dr. Waye. Did you take an impression and thereby obtain a very close joint between the artificial and the natural root?

Dr. Keely. No sir. I once inserted six superior teeth and the antagonism was squarely against the lower teeth. I protested against doing it, but the lady's husband would have it done. They lasted six years. In another case I inserted two incisors and they lasted several years, bearing the whole force of mastication.

Dr. Waye. I most heartily indorse what has been said regarding pivot teeth, especially in reference to plates. I think that a pivot tooth properly inserted is superior to any thing that can be made upon any material and in any manner in the shape of a plate. Particularly as regards rubber plates. The class of dentists who extract single roots in cases like these which have been mentioned, are the very ones, who, as a rule, do not make an elegant denture. They put in a plate which will cover the entire roof of the mouth and is not usually very well fitted about the necks of the teeth, so that the food crowds into the spaces and causes much discomfort, besides interfering with the articulation. The plate is of no use excepting for appearances. I have been in the habit of inserting pivot teeth even where there were only four teeth in front.

My method is to take an impression of the root, strike up a plate and solder to it a pivot which I barb, and then force it up into the root which has been previously filled with Hill's topping. This method leaves very little space for accumulations. It is certainly to be preferred to an atmospheric plate, for single teeth. Teeth are not so much injured by clasps I think, as is generally supposed, excepting where the file is used. I think the mischief is done by the accumulation of food. It is the result of a neglect of scrupulous cleanliness.

The wear on the plate which is nicely adapted is slight. The imperfect adaptation of clasps is no doubt the cause of teeth wearing out. If I were under the necessity of wearing a plate, I should have a clasped gold plate.

Dr. Taylor. I regard the extraction of one, two, three or four good roots, every thing else being right in the mouth —for the purpose of inserting a plate, as mal-practice. I do not see how any man with just regard for his obligations to his patients and to his profession, can do so. These thoughts have forced themselves more and more upon my mind for the last ten years. I am glad to see that the profession is coming to view this matter in the right light.

I wore a pivot-tooth twenty-two years and I would not willingly have exchanged it for the best plate that could be made. I doubt very much whether any individual would wear a single, suction plate tooth. I never could get used to a plate in my mouth—never expect to—and if all persons are like me I am very certain that few persons would wear two or three teeth on such a plate.

Dr. Taylor stated, that he inserted six teeth, carved from the hippopotamus tusk, for a gentleman, which did him good service for nine years,

Dr. Roudebush. I am sorry the practice is not more prevalent. A small plate, bearing one or two teeth, almost always causes dissatisfaction, I know that from a long experience.

Dr. Rehwinkel. I can bring some testimony on the other side. It is claimed that pivot-teeth are not now the style. Some years ago I had occasion to ask some one of these gentlemen in this connection, what the objection was to pivot-teeth. His answer was: "I don't like them." I asked—what would you do in case an intelligent patient persisted in having a tooth pivoted? He answered, "I would say it is not the present style."

Now through all the various changes we have experienced, I have never been brought to believe that a plate is preferable to a well set pivot-tooth. Whenever the condition of the gums will admit of it, I have always regarded this as the nearest conformity to nature. It is possible to set a pivoted crown upon a bicuspid or even a molar, sometimes. By screwing on the crown the stability is increased. In certain cases, where the roots are large enough, they do admirably. In small roots like the lateral incisors, it is not applicable. I rely more upon a gold pivot in such roots, for the reason that the lateral strain upon so small a root is apt to split it, if a wooden pivot is used. A metalic pivot is needed, and a cylinder to protect the root, the cylinder he packs around with amalgam. This operation can be performed upon a lower bicuspid with two roots.

Dr. Taft. I have advocated for years, the retention of healthy roots in the mouth, where there is no special occasion for their extraction; and I even include roots which may be made healthy, although in a diseased condition in many cases. I regard the retention of these as very important. The roots of molar teeth, in a great majority of cases, and in the mouths of all persons who appreciate the value of their natural teeth, I insist upon retaining. Not merely for the purpose of placing plates over them, but there are two or three reasons for it. I appreciate the idea of retaining roots, for the purpose of placing crowns on them. But the preservation of the contours and fullness of the investing parts, is also of primary importance. It is well known that the building out, to supplement lost tissue, by artificial means, is very difficult and often unsatisfactory. The difficulty is best obviated by retaining the roots in the mouth, if possible. The contracted, pinched appearance of the mouth, caused by the shrinkage of the tissues after the roots are extracted, often baffles the efforts of the most pains-taking operator to remedy. There is another reason, and that is the retention in position of teeth which are not decayed. It is very well known to those of extended observation, that teeth frequently change their position when roots adjoining are removed. Another reason is that these roots often serve an important part in mastication. They are better than the gums. After roots are removed, the adjacent teeth are often allowed to remain idle, and as a consequence, suffer. I have often filled the canals of roots and placed a plate over them.

I think, taking all things into account, a good root is half as good as a sound crown, even in molars, and better than an artificial tooth. Many times I have treated diseased roots and restored them to a condition to receive a filling.

Dr. Roudebush spoke of the indispensable necessity of preliminary treatment in most cases of roots before proceeding to pivot them.

Dr. Taft said, that in many cases, salicylic acid could be advantageously used in old roots—forcing it to the canal before inserting the filling.

Dr. M'Kellops. If I understand, you leave the acid in the root?

Dr. Taft. Yes sir.

Dr. Watt. The first pivot-teeth I ever set were worn for sixteen years. I have inserted others which have lasted almost as long, using hickory pivots. In the case of a man seventy years old I once cut off the incisors, bicuspids, cuspids, and on one side, a molar, and placed over them a plate of gold. The roots were filled up with ossific matter, with the exception of two which I filled with gold. This case has done well for five years. There are cases however—as when the roots are affected with granular degeneration, where pivoting is unwise. Better extract at once.

Dr. Roudebush. I have been asked how I would set a pivot tooth when the line of the root canal is not parallel with the line of the perpendicular crown. In that case I pass a gold wire through the hickory and then bend it to suit my purpose.

Dr. Rehwinkel. Dr. Taft, does your method apply to roots of which the pulp has been dead some time, or to cases where the pulp has been devitalized?

Dr. Taft. Ordinarily, in cases of recent removal of the pulp by an operation, I do not use the acid as described. If the pulp has been dead for an indefinite length of time and there is no manifestation of discharge, I treat it at once in the manner described. If there is a discharge, of course it must be arrested first.

Dr. Rehwinkel. In the case of a novice, it is possible that he would not discriminate wisely, in deciding when a root is in condition to be pivoted. The effect of checking the discharge by inserting a pivot and setting it in, will be to cause it to force for itself a new outlet. There will then be an abscess.

When the pulp however is in a numnified condition, as we often find it, it may do perhaps to risk the method that Dr. Taft speaks of, in a healthy subject. But whenever there is a discharge I should not rely upon treatment of that kind, because the antiseptic qualities of the salicylic acid would not be sufficient to overcome the unfavorable condition in the pulp cavity.

Dr. Roudebush. Have you ever seen a case of erysipelas result from mal-treatment in these cases?

Dr. Rehwinkel. I have not. But I am not sure but what it may occur.

Dr. Taft called for some expression as to the merits of the celluloid base.

Dr. Roudebush. Some dentists, high in the profession, have pronounced celluloid an utter failure. I am astonished. I have had no cases which have not given perfect satisfaction. I use dry heat—no steam for me. I use gum teeth usually. It does not affect the mucous membrance as rubber often does, and it does not become discolored very much.

(Dr. Rehwinkel.) It becomes discolored as readily as a meerschaum pipe from the effect of tobacco.

Dr. Roudebush. Do you use steam or dry heat?

Dr. Rehwinkel. Every method that has been suggested I have used. I do not think that the manner of making the plate affects that matter. The stain of tobacco juice may perhaps be removed—not so the stain of smoke. That has been my own experience. I have worn several plates. It shrinks when left out of the mouth. After full consideration and several trials, I am of the opinion that celluloid, as a substitute for the cheap bases, does not answer in all respects. In some cases it will not do at all.

Dr. Berry reported adversely to celluloid

Dr. Cameron agreed with Dr. Rehwinkel's opinion. Has had sucesses and failures. In many cases it is better than rubber—if it were not he would not use it. (Subject passed.)

Dr. Rawls, who was appointed at the meeting in 1876 to prepare a report on replanting teeth, said that he thought that the idea that because the authors of former times did not regard favorably the subject of replanting, there was no merit in it, is a mistake. The method proposed by Dr. Atkinson at Fort Wayne, Indiana, is worthy of consideration. After extracting the tooth, wash the socket with salt water or tincture of myrrh or salicylic acid—fill the root and replace it. Hold the tooth in a napkin saturated with warm water. Remove all spicula of bone from the cavity. If the tooth drops down partly out of the cavity, allow it to do so, that permits the coagulated blood to escape. He has performed the operation successfully in about thirty cases; had but little trouble in the way of pain or swelling.

Dr. Jay asked for advice as to the best means to be used to effect a cure of an abscessed tooth which had resisted persistent treatment.

Dr. Rawls. Extract it, by all means, and pursue the course I have indicated. Probably there is a large sac at the apex which you cannot properly medicate by the ordinary means.

Dr. Jay. I discover, upon introducing a probe through the foramen, that the bone beyond is softened. Now will that diseased bone exfoliate and pass off after the root is extracted and reinserted?

Dr. M'Kellops. Is the bone rough?

Dr. Jay. I am not able to say.

Dr. Rawls was of the opinion that after the tooth was removed and cleansed, the periosteum would recover a healthy tone and a cure be effected.

Dr. M'Kellops extracted an incisor for a sailor who had previously sought relief from a dentist in Memphis. He had drilled through the apex and treated it unsuccessfully. Dr. M'Kellops found it impracticable to treat it satisfactorily in that way, therefore extracted it—filled the root with gutta percha, covering this with a gold plug and reinserted the tooth. In another case, which he treated by injecting creasote for quite a while without effecting a cure, he filled with gutta percha dissolved in chloroform, and in two weeks the cure was complete. It has given no trouble for four years.

Dr. J. Taft. Within the last two years I have been fully convinced of the utility of extraction and reinsertion in many such cases as have been quoted. I regard it as being of the utmost efficiency. Especially where we find granular degeneration of the periosteum. I know cases where the operations have developed no unfavorable after-results, in the course of ten years. I have yet to see the first case of a tooth removed and properly treated, that has given any serious trouble. Since the first of October there have been about fifty operations of this kind performed at the clinics in the Dental College in Ann Arbor. These cases embraced all phases of diseased teeth, from an exposed pulp, hopelessly diseased, to chronic abscesses. Some of the latter cases were of twelve years standing. Only one failure has been reported, and that was no doubt due to the indiscretion of the patient in exposing himself so that he caught cold. In several cases, patients were able to eat on molar teeth three or four days after the operation. The ages of these patients vary from fifteen to seventy-three years.

It does not necessarily follow that the operation cannot succeed because the periosteum is often removed from the root. The socket still remains lined, and the possibility of its performing all the necessary functions is by no means doubtful or remote. Of course the tooth should be extracted with care to avoid laceration of the parts.

Dr. Jay. Have any cases of tetanus ever resulted from these operations?

Dr. Taft. I have heard of no cases. In a majority of cases the patient suffers no pain after leaving the operating room.

Dr. M'Collum I have performed this operation in eighteen cases without a failure.

---

*Thursday Evening, March 8th*, 1877.

---

Fourth subject: "Extraction of the six year Molars—where indicated."

Dr. Smith. When I first entered the profession I was impressed with the idea that I ought to save all the teeth. But observation has taught me that the extraction of these teeth is sometimes best. The indications for it are usually presented a year or two after their eruption, until the age of twelve or fourteen years. If after that age, a good permanent operation can be made, it is well to save them. But prior to that age we must carefully discriminate. There is a large percentage of these teeth which, because of imperfect organization, ought undoubtedly to be extracted. In many cases, expensive operations are performed which have to be performed over again and again. If they decay early, it is often impossible to save them. The fact of their decaying early is an indication of a weakness of organization which renders the probability of their lasting permanently, very slight. It requires a good deal of courage sometimes to perform one's duty and extract them. But after noting the different success of other operators as well as myself, in saving these teeth, I commit myself to the opinion that if defective before about fourteen years of age, they had better be extracted.

Dr. Taft. I do not ordinarily extract these teeth unless they are badly diseased and are productive of injury to surrounding parts. The idea that if these teeth are removed, the second molars will in time fill up the space, has not always been confirmed in my experience. I have extracted these teeth for children of eight and nine years, and the spaces have never been filled up. I often prefer to save them, even if but temporarily in order to insure a proper development of the jaw.

Dr. Smith. Twenty per cent of the operations I make are for persons under fifteen years of age and in first molars. For females, they are in the ratio of sixty to fifty-four males. There is another reason which occurs to me, for extracting the six year molars, and that is that the wisdom teeth are often rendered more servicable in masticating. As it is, the wisdom teeth are fast becoming rudimentary.

Dr. Taft. Had three permanent teeth removed when a boy and the spaces never closed up. He hesitates to subject any one to the risk of the inconvenience that he has suffered in that respect.

Dr. Osmond. Extracts a great many more of these teeth than formerly. Am convinced that such teeth when carious should be extracted about the ninth or tenth year.

Up to the eighth year, would try to save them. If they are extracted later in life, there remains a permanent space oftentimes.

Dr. Clayton. In confirmation of Dr. Smith's opinion that the wisdom teeth are becoming rudimentary, cited the fact that an examination of the skulls of Indians, disclosed the fact that their wisdom teeth were much larger than those we are accustomed to see.

Dr. Smith stated that Dr. Morse, a scientist, had remarked the absence of the lateral incisors in many cases, as an indication of their being omitted entirely in future generations. The fact that they are often seen with a pointed cusp like a canine, seems to indicate that they are becoming rudimentary.

Dr. Keely. In a certain family I know of, only one child out of seven has the lateral incisors.

Would extract the first permanent molars when they are defective and the jaw is crowded. Nevertheless he has been very much perplexed sometimes to know what to do. Protests against the injudicious practice of inserting large gold fillings in these teeth. Tin is better.

When to extract, is a question which has often perplexed him.

Dr. Jay. If indications are favorable would save these teeth for a time at least, until the osseous structure of the jaw is thoroughly developed. Has noticed that in time, these teeth become more dense in structure.

Has heard that giant's skeletons have eighty-four teeth. From this he would infer that there is some foundation for the theory of the superfluous teeth being omitted.

Dr. Taft in reply to a question, answered that it was sometimes expedient to treat these teeth, when aching, and fill the roots. If affected with chronic abscess, it is often better to extract.

Dr. Smith. Would always, in such cases, extract the teeth.

### FRIDAY MORNING SESSION.

Subject: "Filling teeth; comparative value of different materials and different methods of operating."

Dr. Sage. Mentioned a test he had made on a tooth filled *in the mouth* with cohesive foil,—a large contour operation. Immersed the tooth in analine dye for several days and no discoloration about the edges of the filling was apparent. [Note: this tooth was extracted by another dentist at the lady's request, as she objected to the appearance of the gold. The tooth was *not* filled with a view of subjecting it to this test.]

Dr. Berry spoke of the value of os-artificial as a cheap filling. The new method of finishing these fillings with French chalk, he thinks a great improvement.

Dr. Osmond. Has noticed that the harder os-artificial preparations are most easily affected by the chemical action of the oral fluids. He places more reliance upon a filling made of oxide of zinc, pure, mixed with chloride of zinc. The cement Plombe (German cement) has proved more durable in his practice than any of the patented compounds. Those cases in which the calcareous portions of the tooth are dissolved out, leaving the gelatinous portions, offer the least prospect for success with a cement filling. He spoke of Dr. Ludwig's process, which consists in hardening the surface of the os-artificial with powdered French chalk applied with a warm spatula. Has but little faith in it. If the saliva is thick and ropy, he regards the condition as unfavorable for the use of os-artificial.

Dr. Smith. Calcined magnesia powdered and rubbed over the surface of an os-artificial filling will produce as durable a finish as that made with the French chalk. The application must be made while the filling is still very soft. The idea however that this filling is as durable as gold, is utterly absurd.

Dr. M'Kellops. I have a few remarks to make on the methods of filling teeth and wherein I think the failures and successes occur. It is as much a matter of fine instruments and fine workmanship, in many cases, as anything else. It costs but a few dollars more or less to provide oneself with first class instruments in perference to poor ones. Too much care cannot be taken in finishing fillings. The electric mallet is a very valuable assistant. It requires practice to use it to advantage, and some knowledge of the peculiarities of electric batteries is requisite.

After some remarks on the peculiarities of the electric mallet and the methods of manipulation with it, the next subject was announced: "The care of the teeth during gestation and lactation."

Dr. Watt. What is a woman doing when she is raising a child? In the first months of gestation, before the bone formation begins, you would naturally suppose that there is not a much greater demand for the bone phosphate than at other times. But the bone phosphate is essential to the formation of cells. There *is* then some demand on the woman's system. At first the drain is not very heavy. There is usually nausea at first, and the woman neglects her diet and the consequence is she does not accumulate lime salts. A good plan is to have her take her morning meal later—lie in bed until she has an appetite for breakfast, Endeaver to get her into the most perfect condition of health possible.

The artificial treatment with phosphates is often sneered at,—chemical food is sneered at, and yet there is no food used which is not chemical. Now see that the woman has nutritious diet at this early stage. Fresh eggs are rich in phosphates also beefsteak; pork is not. If you burn a piece of pork you will find that the ashes have less of the phosphate in them than any other meat-ashes. The fact that we are a nation of pork eaters accounts in part for our bad teeth. Harm has been done by persuading the people that fresh meats are not healthy. When the bones of the child begin to be formed, there is an increased demand for the lime salts, but there is special wisdom in having a goodly supply stored in the blood in the early months of gestation, to meet the wants of this period. In such cases, you can often do good by co-operating with the family physician.

Dr. Osmond. I fully agree with the remarks of Dr. Watt. We all appreciate the necessity of administering the phosphate of lime and other pabulum.

Dr. Rawls. I believe that the idea of the mother's system being impoverished by the drain to accomplish the formation of the child's bones, is exploded. The theory is that the *process of the formation* of the lime salts, and their appropriation by the fœtus, is interfered with in some way.

Dr. Osmond. I have seen a case where, after devitalizing the pulp and filling the roots, the teeth maintained their structure while teeth with living pulps were softening. That seems to prove that absorption, takes place from within. I make the proposition that the mother is improverished in order to supply the child. The cause I think is this, shortly after she becomes pregnant, the woman begins to neglect exercise. She hides herself during the last three months. The child is born and the parents boast of its weight and its size. Had the mother taken proper exercise the child would have been healthier and lighter in weight. By all means encourage the woman to take exercise during the period of her confinement. She requires more than the usual amount of oxygen in order that the food may be properly assimilated and converted into tissue. The poor teeth of children are often very much bettered by the administration of chemical food. Exercise must be recommended with it. As to nausea, a light repast taken just before the tendency to vomit comes on, will often prevent it.

Dr. Rehwinkel. I am gratified to find that this subject is becoming of universal interest to the profession. This is a matter which directly affects the results of many of our operations. Every one knows the difficulties we labor under in attempting to make our influence felt in this particular matter. A great many ladies would think that a dentist transcended the bound of modesty in broaching this subject to them. That may seem silly, but the fact remains. We may exert our influence directly however by impressing upon the medical faculty the importance of attention to these matters. Dr. Rehwinkel also spoke at length on the pernicious effect of such remedies as muriate of iron and other preparations of iron, as administered by some physicians without respect to their effect upon the teeth.

Dr. C. R. Taft stated that he had often conversed freely about these delicate matters with his lady patients, and had never been repulsed, or seen any indication that the action was regarded as an impertinence. Of course some tact is required to avoid giving offense or shocking the lady's sense of propriety.

Adjourned.

